# Oral health-related quality of life of patients after heart transplantation and those with heart failure is associated with general health-related quality of life: a cross-sectional study

**DOI:** 10.1007/s11136-020-02439-z

**Published:** 2020-02-04

**Authors:** Gerhard Schmalz, Mirjam Eisner, Christian Binner, Justus Wagner, Josephine Rast, Tanja Kottmann, Rainer Haak, Andreas Oberbach, Michael A. Borger, Jens Garbade, Dirk Ziebolz

**Affiliations:** 1 Department of Cariology, Endodontology and Periodontology, University Leipzig, Liebigstr. 12, 04103 Leipzig, Germany; 2 University Department for Cardiac Surgery, Leipzig Heart Center, Leipzig, Germany; 3 CRO Dr. med. Kottmann GmbH & Co. KG, Hamm, Germany; 4 Department of Diagnostics, Fraunhofer Institute for Cell Therapy and Immunology Leipzig, Leipzig, Germany

**Keywords:** Dental care, Heart transplantation, Oral health, Oral-related quality of life

## Abstract

**Purpose:**

The aim of this cross-sectional study was to examine the oral health-related quality of life (OHRQoL) in patients after heart transplantation (HTx) and those with heart failure (HF).

**Methods:**

In total, 186 participants (HTx: 104, HF: 82) were recruited from the University Department for Cardiac Surgery, Leipzig Heart Centre, Germany. OHRQoL was assessed with the German short form of the oral health impact profile (OHIP-G14). Health-related quality of life (HRQoL) was evaluated using the short form 36 survey (SF-36). Furthermore, the dental and periodontal treatment need was recorded.

**Results:**

With an OHIP-G14 sum score of 6.58 ± 6.40 [5; 2.5–8] in the HTx group and 5.54 ± 5.47 [5; 2–7] in the HF group, no clinically relevant or statistically significant difference was apparent (*p* = 0.39). The SF-36 scales for physical functioning, role-physical, general health and vitality were significantly worse in the HF group compared with the HTx group (p_i_ < 0.01). A worse SF-36 physical component summary was significantly associated with a higher OHIP-G14 sum score (HTx: *p* < 0.01, HF: *p* = 0.04). In the HTx group, a significant association was also observed for the mental component summary (*p* < 0.01). Multiple regression analysis revealed physical component summary (*p* = 0.04) and mental component summary (*p* < 0.01) in HTx, and physical component summary (*p* = 0.02), mental component summary (*p* = 0.02) and smoking (*p* < 0.01) as significant predictors for OHIP G14 in HF.

**Conclusion:**

The OHRQoL in HF and HTx patients appears to be mainly associated with general HRQoL. Therefore, multidisciplinary dental care concepts may be recommended to improve oral health conditions in these patients.

## Introduction

Quality of life has developed into a major aspect and treatment outcome with increasing relevance, especially in patients with chronic diseases [1, 2]. Thus, health-related quality of life (HRQoL) is an important factor for patients with cardiovascular diseases, where associations with mortality, cardiovascular events or hospitalization might be apparent [3]. A recent systematic review article on HRQoL of heart-transplanted individuals demonstrated a positive influence of transplantation on HRQoL; however, depression, pain, gastrointestinal symptoms and oral health conditions are potential influential factors of HRQoL in these patients [4].

As a part of general HRQoL, oral health-related quality of life (OHRQoL) can reflect the impact of oral diseases and conditions on patient quality of life [5]. Thus, different dimensions including functional as well as psychosocial impacts of oral health can be assessed [6]. Accordingly, OHRQoL can allow insights into the influence of oral health on the general well-being of patients. Only one study is available that examines the OHRQoL of patients after heart transplantation, and it concluded that there is a low perception regarding the influence of oral health on the quality of life for these patients [7]. However, the available study did not address potential associations with oral health, treatment needs or general HRQoL.

Several studies have investigated OHRQoL and its potential associations with oral diseases in patients with different chronic diseases and conditions, including haemodialysis, rheumatic diseases or solid organ transplantation [8–15]. These previous studies repeatedly show a nearly unaffected or slightly reduced OHRQoL, often irrespective of insufficient oral health situations in these patients [8–13]. It was thereby assumed that general disease-related parameters would affect the OHRQoL in these chronical diseased patients more strongly than oral diseases or treatment needs [11, 12]. Considering the vulnerability of patients after organ transplantation due to their lifelong immunosuppression [16], this situation could be serious. Patients might waive regular dental consultations because of the perception that their oral health is unaffected, even though they may clinically require high dental and/or periodontal treatment needs [12]. This could be of high relevance for dental care and the clinical management of patients after heart transplantation. In this context, comprehensive dental rehabilitation, especially the elimination of infectious foci prior to heart transplantation, appears necessary [17]. Accordingly, differences in oral health should appear between patients after transplantation and potential transplant candidates, such as patients with heart failure. However, it is unknown whether there are differences between these patient groups regarding their oral health conditions and whether transplantation affects individuals’ OHRQoL as well as its relation to their general HRQoL.

Accordingly, the aim of the current cross-sectional study was to comprehensively examine OHRQoL in patients after heart transplantation compared to patients with heart failure. These findings should be interpreted and discussed alongside reference values for a general healthy population as well as results from further studies focused on patients suffering from systemic diseases. Furthermore, the general HRQoL as well as dental and periodontal treatment needs should be assessed and examined with respect to their potential associations with OHRQoL. We hypothesized that patients with heart transplantation would show a nearly unaffected OHRQoL, which would not be associated with oral health conditions but would be related to general HRQoL.

## Methods

This cross-sectional study was executed to compare the OHRQoL of patients with heart failure (HF) and after heart transplantation (HTx). The study protocol obtained approval from the ethics committee of the Medical Faculty of University of Leipzig (No: 414/16-ek). All included participants were informed verbally and in writing and provided their written informed consent.

### Patients

Patients who attended the University Department for Cardiac Surgery, Leipzig Heart Centre, Leipzig, Germany were recruited for the study during their routine follow-up appointment. An age of at least 18 years was defined as the inclusion criterion. Further inclusion criteria were not defined and patients were recruited independent of their time since HTx. If clinical examination was impossible due to a worse general health status or if patients suffered from autoimmune diseases (e.g. rheumatoid arthritis) or infectious diseases (e.g. hepatitis A, B, C, tuberculosis, or HIV), these patients were excluded. Further exclusion criteria were pregnancy as well as being edentulous. After checking the inclusion and exclusion criteria, participants underwent an examination. General and clinical cardiological data, including smoking habits (smoker: currently smoking, former smoker: smoking within five years before examination, and non-smoker: no smoking for at least five years), underlying heart diseases and comorbidities, were elevated from the participants’ medical records. A generally healthy control group was not included.

### Questionnaires

#### Oral Health Impact Profile (OHIP G14)

The German short version of the Oral Health Impact Profile (OHIP G14) was applied to all participants as a valid instrument to assess their OHRQoL [18, 19]. This questionnaire evaluated the presence of 14 functional and psychosocial impacts that participants may have experienced in the previous month resulting from complaints with their teeth, mouth or dentures. For the applied OHIP G14 questions, the five different answer possibilities were as follows: very often = “4”, fairly often = “3”, occasionally = “2”, hardly ever = “1”, and never = “0”. Thus, a total score between “0” (all questions answered with “never”) and “56” (all questions answered with “very often”) could be achieved. For analysis, statistical significance was considered; moreover, following the minimal important difference principle [20], differences in OHIP G14 values of at least 2 points were interpreted as clinically relevant. The sum score of the OHIP G14 values, as well as the two patterns “oral function” and “psychosocial impact”, were analysed [6]. To interpret the OHIP G14 sum score, reference values for generally healthy fully or partially dentate patients were considered from the literature [19]

#### Short Form 36 health survey (SF-36)

The SF-36, consisting of a total of 36 items, was used as a standardized and validated questionnaire to assess the HRQoL [21]. In the current study, a German translation was applied for the participants [22]. Thus, different scales, including physical functioning, role-physical, general health, vitality, bodily pain, social functioning, role-emotional and mental health, were analysed. Of these scales, two distinct higher-ordered clusters, the physical component summary (PCS) and mental component summary (MCS) measures, were developed for further analysis. Data from the scales are presented as raw values (0–100). For interpretation, higher values represent a better HRQoL.

### Oral examination

At the Department of Cardiothoracic Surgery, Leipzig Heart Centre, Leipzig, Germany, an oral examination was executed by two experienced and calibrated dentists (kappa > 0.8) using standardized conditions. Participants received an antibiotic prophylaxis (2 g amoxicillin or clindamycin) according to recent guidelines before the oral examination [23]. Both dental and periodontal examination were executed.

During the dental examination, decayed- (D-T), missing- (M-T) and filled-teeth (F-T) indices (DMF-T) were assessed visually with a mirror and probe in accordance with the WHO [24]. In the case of carious lesions requiring invasive dental intervention (D-T > 0), the dental treatment need was evaluated. Periodontal probing depth (PPD) and clinical attachment loss (CAL) were evaluated with a periodontal probe (PCP 15, Hu-Friedy, Chicago, IL, USA). The definition of periodontal treatment need was defined by the presence of a periodontal probing depth ≥ 3.5 mm in at least two different sextants according to the periodontal-screening index [25, 26]. If dental and/or periodontal treatment needs were present, the overall dental treatment need was rated.

### Statistical analysis

The statistical analysis was performed with SPSS for Windows, version 24.0 (SPSS Inc., US). The metric variables were tested for their normal distribution with the Kolmogorov–Smirnov test. The analysis of SF-36 was conducted with official analysis software (Hogrefe GmbH & Co. KG, Goettingen, Germany). A t test was applied to compare two independent, normal distributed samples. In the case of homogeneity (Levene test), the student’s *t *test was used. Non-normally distributed samples were analysed with the Mann–Whitney *U* test. For the comparison of more than two independent, non-normally distributed samples, the Kruskal–Wallis test was performed. Categorical data were analysed using the Chi-square or Fisher test. For multivariate analysis, a multiple regression with backward elimination was executed. Two-sided significance testing was used for all the applied analyses, where the significance level was set to *p* < 0.05.

## Results

### Patients

In total, 186 participants (HTx: 104, HF: 82) with a comparable age (*p* = 0.77) and gender (*p* = 0.15) distribution were included in the study (Table 1). The number of smokers was significantly higher among the HF participants (*p* = 0.03). Some significant differences in the underlying heart disease and comorbidities are presented in Table 1.

**Table 1:** Patient characteristics

	HTx (*n* = 104)	HF (*n* = 82)	*p* value
Gender (male in % [*n*])	75% [78]	84.1% [69]	0.15
Age in years (mv ± sd)	55.26 ± 12.16	54.90 ± 11.14	0.77
Time since HTx in years (mv ± sd)	6.8 ± 5.16	–	–
Smoking habits % [*n*]			
Smoker	4.8% [5]	16% [13]	**0.03**
Non-smoker	76% [79]	63% [51]	
Former smoker	19.2% [20]	21% [17]	
Underlying heart disease % [*n*]			
DCM	62.5% [65]	63.4% [52]	0.99
ICM	29.8% [31]	31.7% [26]	0.87
Valvular insufficiency	12.5% [13]	40.2% [33]	** < 0.01**
Atrial fibrillation	6.7% [7]	29.3% [24]	** < 0.01**
Comorbidities % [*n*]			
Hypertension	56.3% [58]	74.4% [61]	**0.01**
Diabetes mellitus	31.7% [33]	35.4% [29]	0.64
Osteoporosis	4.8% [5]	4.9% [4]	0.99
Renal insufficiency	83.7% [87]	45.1% [37]	** < 0.01**
Obesity	50% [52]	42.7% [35]	0.38
NYHA-class			
I	–	6% [4/69]	–
I–II		6% [4/69]	
II		43% [30/69]	
II–III		6% [4/69]	
III		29% [20/69]	
III–IV		4% [3/69]	
IV		6% [4/69]	

Significance level: *p* < 0.05

Significant results (*p* < 0.05) are highlighted in bold

*HTx* heart transplantation, *HF* heart failure, *dcm* dilated cardiomyopathy, *icm* ischaemic cardiomyopathy, *mv* mean value, *sd* standard deviation, *NYHA* New York Heart Association

### Oral health and treatment needs

The dental health parameters M-T and DMF-T were comparable between the HTx and HF participants (*p* > 0.05). Similarly, dental and overall treatment needs did not significantly differ between the two groups (*p* > 0.05). Only the periodontal treatment need was slightly higher in HF participants compared to HTx participants (95.1% vs. 85.6%, *p* = 0.05; Table 2).

**Table 2:** Results of the dental findings and OHIP G14 scores between groups

Parameter	HTx (*n* = 104)	HF (*n* = 82)	*p* value
DMF-T (mv ± sd)	16.08 ± 7.11	16.90 ± 6.66	0.46
M-T (mv ± sd)	6.90 ± 7.27	7.32 ± 7.64	0.71
Dental treatment need % [*n*]	16.3% [17]	17.1% [14]	0.99
Periodontal treatment need % [*n*]	85.6% [89]	95.1% [78]	**0.05**
Overall treatment need % [*n*]	86.5% [90]	95.1% [78]	0.08
OHIP G14 sum score	6.58 ± 6.40 [5; 2.5–8]	5.54 ± 5.47 [5; 2–7]	0.39
OHIP G14 pattern psychosocial impact	2.04 ± 3.86 [0; 0–3]	1.26 ± 3.53 [0; 0–2]	0.24
OHIP G14 pattern oral function	1.30 ± 2.40 [0; 0–2]	0.78 ± 1.68 [0; 0–0]	**0.04**

Significance level: *p* < 0.05. Significant results (*p* < 0.05) are highlighted in bold

*HTx* heart transplantation, *HF* heart failure, *mv* mean value, *sd* standard deviation, *DMF-T* decayed-, missing- and filled-teeth index, *M-T* missing teeth, *OHIP* oral health impact profile. OHIP values are given as the mean value ± standard deviation [median; 25th–75th percentile]

### OHIP G14 values

Based on a sum score of 6.58 ± 6.40 [5; 2.5–8] in the HTx group and 5.54 ± 5.47 [5; 2–7] in the HF group, no clinically relevant or statistically significant differences were apparent (*p* = 0.39). While the pattern of psychosocial impact (HTx: 2.04 ± 3.86 [0; 0–3], HF: 1.26 ± 3.53 [0; 0–2], *p* = 0.24) was comparable between the groups, a statistically significant difference was observed for the pattern of oral function (HTx: 1.30 ± 2.40 [0; 0–2], HF: 0.78 ± 1.68 [0; 0–0], *p* = 0.04; Table 2). This difference was not clinically relevant, in accordance with Reissmann et al. [19]. Several differences were found for singular items in the OHIP G14 questionnaire, including “feeling of tension” (*p* = 0.03), “interrupting meals” (*p* = 0.04) and “difficulty performing jobs” (*p* < 0.02). The distribution for the singular items is presented in Table 3.

**Table 3:** Results of the periodontitis questionnaire

Question [*n*]	Group	Point Score					*p *value
		Never (rating 0)	Rarely (rating 1)	Sometimes (rating 2)	Often (rating 3)	Very often (rating 4)	
Trouble pronouncing	HTx	88	11	3	2	0	0.88
	HF	70	6	3	2	0	
Taste worsened	HTx	80	12	10	1	1	0.21
	HF	71	6	2	1	0	
Life less satisfying	HTx	83	11	6	2	2	0.71
	HF	64	11	2	3	1	
Difficult to relax	HTx	81	12	6	3	2	0.64
	HF	70	6	2	2	1	
Feeling of tension	HTx	83	11	7	3	0	**0.03**
	HF	76	2	2	0	1	
Interrupting meals	HTx	88	10	6	0	0	**0.04**
	HF	77	4	0	0	0	
Uncomfortable to eat	HTx	83	12	6	2	0	0.10
	HF	70	3	8	0	0	
Short tempered	HTx	84	14	3	2	0	0.22
	HF	73	5	2	0	1	
Difficulty performing jobs	HTx	84	10	8	1	0	**0.02**
	HF	76	0	4	0	1	
Unable to function	HTx	89	11	2	2	0	0.10
	HF	77	2	1	0	1	
Embarrassed	HTx	87	13	1	3	0	0.12
	HF	74	4	2	0	1	
Diet unsatisfactory	HTx	89	10	4	1	0	0.31
	HF	75	4	1	0	1	
Oral pain	HTx	83	10	10	1	0	0.79
	HF	66	12	4	0	0	
Sense of uncertainty	HTx	83	4	6	6	5	0.23
	HF	61	3	0	4	3	

Significance level: *p* < 0.05. Significant results (*p* < 0.05) are highlighted in bold

*HTx* heart transplantation, *HF* heart failure

### SF-36 values

The scales for physical functioning (HTx: 68.83 ± 27.83 vs. HF: 51.29 ± 28.61, *p* < 0.01), role-physical (61.08 ± 43.95 vs. 38.27 ± 42.95, *p* < 0.01), general health (55.08 ± 22.64 vs. 44.48 ± 17.98, *p* < 0.01) and vitality (58.35 ± 20.18 vs. 47.70 ± 19.45, *p* < 0.01) were significantly worse in the HF group than in the HTx group. Of the two higher-ordered clusters, the PCS was significantly worse in the HF group than in the HTx group (43.04 ± 11.28 vs. 37.41 ± 11.09, *p* < 0.01; Table 4).

**Table 4:** SF 36 between groups

Parameter	HTx (*n* = 104)	HF (*n* = 82)	*p* value
SF-36 physical functioning (mv ± sd)	68.83 ± 27.83	51.29 ± 28.61	** < 0.01**
SF-36 role-physical (mv ± sd)	61.08 ± 43.95	38.27 ± 42.95	** < 0.01**
SF-36 general health (mv ± sd)	55.08 ± 22.64	44.48 ± 17.98	** < 0.01**
SF-36 vitality (mv ± sd)	58.35 ± 20.18	47.70 ± 19.45	** < 0.01**
SF-36 bodily pain (mv ± sd)	70.23 ± 31.46	76.95 ± 26.94	0.22
SF-36 social functioning (mv ± sd)	78.52 ± 26.05	82.97 ± 22.61	0.39
SF-36 role-emotional (mv ± sd)	76.70 ± 40.38	69.17 ± 43.98	0.25
SF-36 mental health (mv ± sd)	71.26 ± 20.64	69.49 ± 17.69	0.34
Physical component summary (mv ± sd)	43.04 ± 11.28	37.41 ± 11.09	** < 0.01**
Mental component summary (mv ± sd)	49.99 ± 10.55	50.36 ± 9.77	0.89

Significance level: *p* < 0.05. Significant results (*p* < 0.05) are highlighted in bold

*HTx* heart transplantation, *HF* heart failure, *mv* mean value, *sd* standard deviation, *SF-36* short form 36 survey

### Associations between OHIP G14 and oral health as well as HRQoL

In both groups, HF and HTx, a worse SF-36 PCS was significantly associated with a higher OHIP G14 sum score (HTx: *p* < 0.01, HF: *p* = 0.04). In the HTx group, a significant association was also found for the MCS (*p* < 0.01). In contrast, only a trend for an association between the MCS and OHIP G14 sum score was found for the HF group (*p* = 0.06, Table 5). Of the examined oral health parameters, only periodontal treatment need was found to be associated with the OHIP G14 sum score in the HF group (*p* = 0.02; Table 6). In HTx group, the multiple regression analysis of OHIP G14 findings revealed significant results for the PCS (*β* = − 0.242 CI_95_ − 0.267 to − 0.008; *p* = 0.04) and MCS of SF-36 (*β* = − 0.362 CI_95_ − 0.340 to − 0.100; *p* < 0.01). Within HF group, PCS (*β* = − 0.300 CI_95_ − 0.275 to − 0.024; *p* = 0.02), MCS (*β* = − 0.247 CI_95_ − 0.253 to − 0.025; *p* = 0.02) and the presence of smoking habits (*β* = − 0.407 CI_95_ − 7.289 to − 1.958; *p* < 0.01) were found to be significant predictors for OHIP G14 (Table 7).

**Table 5:** Associations of the physical component summary (PCS) and mental component summary (MCS) with the OHIP G14 sum score, psychosocial impact and oral function

OHIP G14		HTx		HF	
		PCS	MCS	PCS	MCS
Sum score	≤ 2	48.61 ± 9.02	54.13 ± 8.66	43.45 ± 9.53	49.28 ± 10.87
	3–5	45.21 ± 9.68	50.93 ± 8.10	35.56 ± 10.97	53.48 ± 7.87
	6–7	38.30 ± 14.64	53.11 ± 8.56	34.88 ± 10.85	50.01 ± 12.35
	≥ 8	38.55 ± 10.32	43.67 ± 12.92	35.16 ± 11.41	45.79 ± 9.44
	*p* value	** < 0.01**	** < 0.01**	**0.04**	0.06
Psychosocial impact	0	44.50 ± 11.35	51.34 ± 9.42	38.28 ± 10.85	51.32 ± 9.41
	1	39.50 ± 12.69	52.85 ± 14.85	35.17 ± 9.49	48.61 ± 11.12
	≥ 2	40.71 ± 10.36	45.79 ± 10.77	35.75 ± 14.67	47.51 ± 9.94
	*p *value	0.16	**0.03**	0.48	0.35
Oral function	0	48.81 ± 8.58	51.62 ± 8.71	38.89 ± 11.08	50.83 ± 9.56
	1	49.61 ± 6.54	57.58 ± 5.44	34.49 ± 7.05	48.98 ± 6.56
	≥ 2	35.90 ± 11.44	42.77 ± 12.04	32.67 ± 10.97	48.97 ± 11.48
	*P *value	** < 0.01**	** < 0.01**	0.13	0.82

Significant findings (*p* < 0.05) are highlighted in bold

*OHIP* oral health impact profile, *HTx* heart transplantation, *HF* heart failure

**Table 6:** Associations between the OHIP G14 sum score and oral health parameters in the HTx and HF groups

Variable	HTx		HF	
	OHIP G14 sum score	*p* value	OHIP G14 sum score	*p *value
DMF-T				
≤ 12	5.59 ± 6.03	0.95	6.00 ± 7.98	0.86
13–16	5.81 ± 4.85		4.93 ± 3.69	
17–21	6.60 ± 7.02		5.23 ± 4.42	
≥ 22	6.56 ± 6.01		5.84 ± 4.35	
M-T				
≤ 1	5.27 ± 4.39	0.47	4.36 ± 3.36	0.25
2–4	6.49 ± 7.22		7.53 ± 9.04	
5–10	5.23 ± 4.59		4.36 ± 3.36	
≥ 11	7.70 ± 7.40		6.38 ± 4.99	
Dental treatment need				
Yes	6.97 ± 5.92	0.18	6.14 ± 3.46	0.19
No	5.59 ± 6.03		5.41 ± 5.81	
Periodontal treatment need				
Yes	6.03 ± 5.67	0.52	5.76 ± 5.50	**0.02**
No	8.64 ± 6.89		1.25 ± 2.50	

Significant results (*p* < 0.05) are highlighted in bold

*HTx* heart transplantation, *HF* heart failure, *OHIP* oral health impact profile, *DMF-T* decayed-, missing- and filled-teeth index, *M-T* missing teeth

**Table 7:** Multiple regression analysis for OHIP findings with regard to age, gender, smoking habits, SF-36 and oral health findings

Parameter	HTx group			HF group		
	*β*	CI_95_	*p *value	*β*	CI_95_	*p *value
SF-36 physical component summary	− 0.242	− 2.67 to − 0.008	**0.04**	− 0.300	− 0.275 to − 0.024	**0.02**
SF-36 mental component summary	− 0.362	− 0.34 to − 0.100	** < 0.01**	− 0.247	− 0.253 to − 0.025	**0.02**
M-T	0.145	− 0.129 to 0.385	0.33	0.204	− 0.021 to 0.318	0.09
DMF-T	− 0.046	− 0.291 to 0.209	0.76	− 0.128	− 0.358 to 0.147	0.41
Dental treatment need	0.024	− 2.808 to 3.616	0.80	0.040	− 2.587 to 3.729	0.72
Periodontal treatment need	− 0.080	− 5.205 to 2.337	0.45	0.122	− 2.296 to 8.389	0.26
Age	− 0.043	− 0.131 to 0.086	0.68	− 0.217	− 0.231 to 0.017	0.09
Gender	− 0.036	− 3.386 to 2.338	0.72	0.021	− 3.215 to 3.907	0.85
Smoking	0.065	− 1.945 to 3.919	0.51	− 0.407	− 7.289 to − 1.958	** < 0.01**

Significant values (*p* < 0.05) are highlighted in bold

*CI* confidence interval, *HTx* heart transplantation, *HF* heart failure, *SF-36* short form 36 survey, *OHIP* oral health impact profile, *M-T* number of missing teeth, *DMF-T* decayed-, missing- and filled-teeth index

## Discussion

### Summary of the main results

Oral health and OHRQoL were nearly comparable between HTx and HF patients. The periodontal treatment need was slightly higher in HF patients and was associated with OHRQoL in this patient group. The HRQoL assessed with SF-36 was worse in the HF group than in the HTx group. OHRQoL was slightly reduced in both groups, and primarily associated with the main scales for the HRQoL.

### Comparison with published data

The focus of this cross-sectional study was to assess the OHRQoL of patients with HF and after HTx. The standardized and validated questionnaire, which was applied to assess the OHRQoL, allows a comparison of the current study’s results with reference values for healthy individuals [19]. OHIP G14 scores between 0 and 4 were previously reported as a reference for generally healthy, fully or partially dentate individuals [19]. This range in OHIP G14 score can be used for interpretation of the current study’s findings. The detected values of 6.58 (HTx) and 5.54 (HF) are slightly higher than the defined upper limit of the reference OHIP G14 score at 4. Therefore, the OHIP G14 sum scores and the overall OHRQoL of the HTx and HF patients can be interpreted as nearly comparable to a generally healthy population. Only one study had previously examined the OHRQoL of HTx patients, and it also showed a slight effect on the OHRQoL; however, the OHIP 49 questionnaire was used in the previous examination, making comparison of the values difficult [7]. Other previous studies performed on patients with solid organ transplantation showed slightly lower OHIP G14 scores compared to the current study; these studies focused on patients after kidney (2.54), lung (1.7) and liver (4.1) transplantation [8–10]. Without further results for patients with HF or organ transplantation, several other German patient groups with chronic systemic diseases might be considered to interpret the current study’s findings. For example, patients undergoing chronic haemodialysis showed a slightly better OHIP score (4.17) than the HF and HTx patients in the current study [12]. Furthermore, previous examinations of patients with rheumatoid arthritis (7.3) and ankylosing spondylitis (6.2) showed slightly reduced OHRQoL that were comparable to those of the HF and HTx patients [11, 13]. Accordingly, the results of the patients in the current study match the unaffected or slightly reduced OHRQoL of patients with chronic diseases and conditions. Thus, the scale of reduction of the OHRQoL in these patients is far smaller than that for generally healthy patients who suffer from oral diseases such as generalized periodontitis or temporomandibular disorders [27, 28].

A further similarity to the available literature is the missing association between oral health and OHRQoL for the patients within the current study, which has been repeatedly described and was hypothesized accordingly [8–15]. The nearly unaffected OHRQoL of the HTx and HF patients despite the high prevalence of periodontal treatment need in the current study is conspicuous, especially considering the effects of periodontitis on OHRQoL in generally healthy individuals [27]. Thus, the periodontal treatment need of the general population in Germany has to be mentioned. With a periodontal treatment need of 75.4% in the age group from 65 to 74 years, which is the most comparable to the current study’s patient age, the HTx (85.6%) and HF (95.1%) groups suffered from a higher periodontal treatment need than the generally healthy population [29]. The observed difference between the HTx and HF groups only hints at performing dental therapy before transplantation in accordance with the demand in the literature [17], but the high prevalence of treatment need in the HTx group argues against sufficient pre-transplant dental rehabilitation. A significant association was detected for the HF group for periodontal treatment need for OHRQoL. The very high prevalence of periodontal treatment need (95%) within the HF group is obvious. The fact that only 4 participants in the HF group did not show periodontal treatment need might limit the potential to draw a robust conclusion from this finding.

The absence of further associations between insufficient oral health and OHRQoL could be serious, because periodontal inflammation is associated with an increased risk of bacteraemia [30], which might be a risk factor for immunosuppressed organ-transplanted individuals [17, 31]. If patients do not feel affected by this oral situation, as potentially reflected by the sufficient OHRQoL, they might waive dental consults due to a lack of complaints. This might explain the insufficient periodontal care situation and the lack of dental management of the patients. Moreover, this might point to a lack of compliance, which would be supported by the fact that current smokers were present in the HTx group. Nevertheless, the missing effect of oral health on OHRQoL measured by the OHIP G14 questionnaire could also reflect the limited validity of the applied questions to a cohort of patients with severe chronic general diseases. This is supported by the fact that previously examined cohorts of patients with chronic general diseases show nearly unaffected OHRQoL as measured by OHIP G14 [8–13], indicating that other questionnaires might fit better. Different example, such as the Geriatric/General Oral Health Assessment Index (GOHAI) for the elderly [32], the Mouth Handicap in Systemic Sclerosis (MHISS) for patients with systemic sclerosis [33] or the Xerostomia Quality of Life Scale (XeQoLS) for patients with Sjörgen Syndrome [34] are available. Accordingly, a specific questionnaire could be composed and applied, which may more likely reflect the subjectively perceived OHRQoL of patients with general diseases such as HF and HTx.

A further point of view is the consideration of the general HRQoL in this context. The applied SF-36 survey is a valid instrument for the assessment of HRQoL in both healthy individuals and patients with heart diseases [3, 35]. First, the HRQoL findings from the current study can be viewed in the context of available reference values. For healthy individuals, average values of 50 ± 10 can be assumed [21]; there are also reference values available for German patients with heart failure [3]. The mean values for the PCS of 42.8 ± 9.3 and MCS of 46.6 ± 10.8 are given in the literature [3]. Accordingly, only the HF group showed slightly lower PCS values compared to the available reference. In the comparison of the two groups, HF patients were observed to present a worse HRQoL than HTx patients. This appears to be in line with the available literature, where an improvement is reported in the HRQoL after transplantation [4]. However, the fear of negative effects and physical limitations are still of relevance for patients after transplantation [36].

As included in the definition of FDI by the Word Dental Federation, the OHRQoL and psychosocial well-being of oral situations is a mandatory part of oral health [37]. Oral health and quality of life are in a complex interrelationship; individual perception of oral conditions is determined by different individual experiences and general concerns [36]. OHRQoL must be seen as a part of general HRQoL [5], resulting in a bidirectional interrelationship in which OHRQoL can affect HRQoL and vice versa. Thus, the impact of oral health on OHRQoL is generally more pronounced than its influence on general HRQoL [38]. Accordingly, the associations found in the current study are somewhat surprising because HRQoL appears to be more strongly affecting OHRQoL than oral health situations in the HF and HTx patients. These findings confirm the previously formulated presumption that general disease or disease-specific parameters would be the main influencing factor on the OHRQoL of patients with severe chronic diseases and conditions [11, 12]. In both the HF and HTx groups, an association between OHIP and the SF-36 PCS was found, while the MCS was just significantly associated with the OHRQoL in the HTx group. Although significance was missed in the HF group, a trend was detected (*p* = 0.06), while the smaller number of HF cases affected this issue. Multiple regression analysis confirmed the relationship between OHIP G14 values and SF-36 PCS and MCS. It is known that regularly OHIP values are related to both physical and mental aspects of the HRQoL [38]. Generally, the increased psychological burden, especially the occurrence of depression and anxiety in patients suffering from HF [39] as well as the limited physical functional capacity [40] might be relevant factors. These limitations of daily life and well-being might be an influential factor for both, general and OHRQoL. This could be a possible explanation for their correlation. Data for patients with heart failure are not yet available. However, this has only been reported for patients with other severe general diseases, such as systemic sclerosis [41]. Considering the association between the psychosocial impact and MCS in the HTx group, the findings could indicate a higher reflection of potential psychosocial factors if they are present in HTx patients. This could exert a greater influence in the HTx group because these patients generally present a better HRQoL than the HF group. The effect on the HF group might therefore be blurred by a worse HRQoL. However, this remains speculative.

Altogether, these results support the necessity for an interdisciplinary understanding of oral health conditions in the context of general health and quality of life. In the case of HF and HTx patients, multidisciplinary approaches to a special dental care programme appear necessary. This recommendation is primarily derived from the fact that insufficient oral health situations are not reflected by the patient’s individually experienced oral conditions and its impact on OHRQoL. This situation explains the reduced oral behaviours, e.g. the low use of additional oral hygiene aids or complaint-oriented dental consultations. Therefore, sensitization and information for the importance of oral health considering the general health and potential psychological co-factors seems recommendable for this vulnerable patient group. Thus, the complex interdisciplinary understanding of the oral health conditions in general health and quality of life based on the specific patient case appears of clinical importance. Moreover, changes in the German health care system, such as cost-free dental care, especially regarding prevention measures for patients with severe systemic diseases, could be a practical approach to improve their dental care situation.

### Strengths and limitations

This is the first study that examined OHRQoL and its potential association with HRQoL as well as oral health in patients with HF and those after HTx. The inclusion of 186 participants and the comprehensive examination is a further strength. The heterogeneity of the study groups with differences in smoking habits, comorbidities and underlying disease are potential limitations. In this regard, the time since HTx might be of relevance because changes in behaviour and oral hygiene with increasing time after HTx might be conceivable. All of the potential participants were recruited irrespective of their time since HTx; this might increase the heterogeneity of the results and limit the conclusions of the study. Furthermore, a longitudinal study design would be necessary to draw causative conclusions; for example, the fact that different patients before (HF) and after HTx were examined does not allow a statement about any potential improvements in the quality of life. Therefore, future studies should focus on these issues and consider the variety of potential influencing factors on OHRQoL. Thereby, the focus was set on OHRQoL and potential predictors in HF and HTx. From clinical perspective, it would also be of interest how good oral health care can help maintain and improve physical and mental quality of life. Because this issue goes beyond the focus of the current study, this could be recognized in future examinations in the field. The applied surveys, OHIP G14 and SF-36, are valid and reference values are available in the literature. Nevertheless, the absence of a healthy control group remains a limitation. The recruitment of a generally healthy control group was omitted based on the following rationales: first, reference values exist for OHIP G14 and SF-36 in the general population and for SF-36 among individuals with heart diseases [3, 19, 21]. Second, it is well known that in regular case dental caries and periodontitis negatively affect the OHRQoL [27, 42]. Third, to interpret oral findings, the Fifth German oral health study can be considered a representative study for the healthy German general population if necessary [19]. Therefore, the absence of a control group appears to be a negligible limitation.

## Conclusion

Patients with HF and after HTx showed a slightly affected OHRQoL, which appeared mainly independent of their insufficient oral conditions. A worse HRQoL was observed for HF patients compared with HTx patients, which was also associated with OHRQoL in both groups. Thus, a multidisciplinary approach that considers different oral and general health-related parameters and provides a special dental care programme is recommendable for patients with HF and after HTx.
